# The preimaginal stages of *Galerita ruficollis* Dejean, 1825 and the position of the tribe Galeritini in the classification of ground beetles (Coleoptera, Carabidae)

**DOI:** 10.3897/zookeys.1044.63085

**Published:** 2021-06-16

**Authors:** Kirill V. Makarov, Andrey V. Matalin

**Affiliations:** 1 Moscow State Pedagogical University, Institute of Biology & Chemistry, Zoology & Ecology Department, Kibalchicha str. 6, build. 3, Moscow 129164, Russia Moscow State Pedagogical University Moscow Russia; 2 Pirogov Russian National Research Medical University, Biology Department, Ostrovitianova Str. 1, 117997 Moscow, Russia Pirogov Russian National Research Medical University Moscow Russia

**Keywords:** Carabidae, development, egg, *
Galerita
*, morphology, mud cells, phylogenetic relationships, preimaginal stages

## Abstract

The complete development cycle of Galerita (Galerita) ruficollis Dejean, 1825 was studied for the first time. In laboratory, at a temperature of 22 °C and long-day conditions, the development from egg to adult lasted 58–60 days. The development of the third instar larva lasted particularly long (on average, 19 days), and the most intense increase in biomass (from 20 to 100 mg) was observed at that phase as well. The extended embryonic development (11–20 days) and the relatively short development time of the third instar larva were found to be characteristic of *G. ruficollis*. The bifurcated protrusion of the anterior edge of the head was proven to represent an outgrowth of the frontal sclerite (frontale), but not of the nasale, as believed previously. The chaetotaxy of *Galerita* larvae is described in detail for the first time.

Based on larval features, the monophyly of the Galeritini + Dryptini group is confirmed. Based on the morphology of the larvae and pupae, this group can be suggested as occupying a separate position within the Truncatipennia, possibly being related to the assemblage that includes Pterostichini, Harpalini, Licinini, Chlaenini, and Platynini. The monophyly of Zuphiitae (sensu Erwin and Sims 1984; Erwin 1985) and the Zuphiitae clade (sensu Ober and Maddison 2008) is confirmed.

## Introduction

The larvae of ground beetles have been studied for almost 200 years, and at present, they have been described from all continents except Antarctica. However, the degree of our knowledge is still extremely patchy and limited. The preimaginal stages of ground beetles of the temperate zones of Eurasia and North America have been investigated most fully, while information on the Carabidae larvae of the tropical and subtropical regions of both the Old and the New World is especially fragmentary. For many genera, the larvae of only single species have been described, often very formally, and for some of them no preimaginal stages are known.

A significant contribution to the knowledge of the larvae of Carabidae was made by Terry Irwin, who was the first to describe the larvae from such genera as *Brachinus* Weber, 1801 (Erwin 1966, 1967), *Eurycoleus* Chaudoir, 1848 (Erwin 1975); *Enceladus* Bonelli, 1813 (Erwin 1978), *Agra* Fabricius, 1801 (Arndt et al. 2001), *Askalaphium* Liebke, 1938 (Erwin and Medina 2003), *Pheropsophus* Solier, 1833 and *Stenaptinus* Maindron, 1906 (Frank et al. 2009), *Leptotrachelus* Latreille, 1829 (Erwin and White 2012). He also pioneered the publication of a synopsis of the immature stages of the tribe Pseudomorphini (Erwin 1981).

The larvae of the tribe Galeritini, which includes the subtribes Planetina and Galeritina, with one and four genera, respectively (Ball 1985a), are amongst the most poorly studied Carabidae. The genus *Galerita* Fabricius, 1801, which includes more than 110 species, shows the highest diversity (Bousquet 2012; Hovorka 2012, 2016, 2017, 2019). Based on adult features, the taxonomy of the tribe has been developed quite satisfactorily (Reichardt 1965, 1967; Ball and Nimmo 1983; Ball 1985a, b; Hunting 2008), while information on the preimaginal stages is incomplete in many ways. Despite the characteristic, unmistakable, and easily recognizable habitus of the Galeritini larvae, information concerning them is very scant. Third instar larvae of nine species of *Galerita* (Sallé 1846; Candèze 1861; Hubbard 1875; van Emden 1942; Kawada 1959; Thompson 1979; Kirk 1980; Costa et al. 1988; Dajoz 2005; Bousquet 2010) and of *Trichognathus marginipennis* Latreille, 1829 (Arndt and Drechsel 1998; dos Santos 2012), as well as pupae of three *Galerita* species (Sallé 1846; Schaupp 1882; Costa et al. 1988; Dajoz 2005) and of *T. marginipennis* (dos Santos 2012) have been described, albeit with a varying degree of detail. The specific features of chaetotaxy in Galeritini larvae have only briefly been mentioned (Arndt and Drechsel 1998; Bousquet 2010).

All preimaginal stages of Galerita (Galerita) ruficollis Dejean, 1825 have been described for the first time, and the taxonomic position of the tribe Galeritini is discussed based on larval features.

## Materials and methods

### Collecting and rearing

On 8 April 2018, 28 specimens of *G. ruficollis* were collected from near the town of Viñales, Pinar del Rio Province, Cuba (22°37'05"N, 83°44'03"W (DMS), 120 m a.s.l.), by Igor Melnik, and then transferred to the laboratory in Moscow, Russia.

From mid-May to mid-December 2018, adults, eggs, larvae, and pupae were maintained under long-day conditions (LD) (16:8) at 22–24 °C and at 75–80% humidity. The beetles were contained in plastic cages of 500 ml capacity (17 × 12 cm). The eggs and the first instar larvae were incubated in Petri dishes 55 mm in diameter, while the second and third instars, as well as the pupae, were maintained in plastic cages of 250 ml capacity (10 × 7 cm). In all cases, coconut chips were used as substrate. In addition, large grains of the loamy soil, pieces of wood, rotten leaves, and green stems of *Polytrichum* sp. were administered to the containers holding adults. Various combinations of substrate components were used to test the effect of substrate quality on egg-laying. For the study of the group effect on the success of oviposition, seven male-female combinations were tested: one male + one female; one male + two females; two males + one female; three males + three females; four males + five females; seven males + eight females, 13 males + 15 females. Both adults and larvae were fed with pieces of larvae of *Zophobas morio* Fabricius, 1776 (Coleoptera, Tenebrionidae), as well as of small cockroaches, aphids, ants, pieces of earthworms, and some other insects.

In total, 49 first instar larvae including two exuviae, 14 second instar larvae including six exuviae, nine third instar larvae including two exuviae, as well as two pupae were studied. All larvae, their exuviae, pupae, and adults are stored in the collections of the Zoology & Ecology Department of Moscow State Pedagogical University, Russia (**MSPU**).

### Measurements

The measurements were taken using an ocular-micrometer mounted on a MBS1 (Lomo) stereo microscope. Eggs, larvae, pupae, and adults were weighed every three days utilising a CAUX120 electronic balance to an accuracy of 0.1 mg.

### Imaging and larval descriptions

Specimens were examined under Leica M165C stereomicroscope or a Zeiss Axio Scope.A1 microscope and photographed either with a Canon EOS 5D Mark III camera with a Canon MP-E 65 mm macro lens or with a Canon EOS 6D camera attached to a Zeiss Axio Scope.A1 microscope. In both cases, the extended focus technique was applied, and photographs were processed using Zerene Stacker software.

The nomenclature of the primary setae and pores follows Bousquet and Goulet (1984) with modifications (Makarov 1996), the numeration of the secondary setae is after Bousquet (1985). The chaetotaxy of Galeritini is highly differentiated and complex due to numerous secondary elements already in the first instar larva. To simplify the presentation, the characteristic of the basic elements of the chaetotaxy is as follows. Macrosetae: type **MA1** – thin, weakly sclerotized, mainly mechanoreceptors (Fig. 6J); type **MA2** – very long and slender, with a strongly developed collar, like trichobothria (Fig. 6K); type **MB** – strong and thick, with a sharp tip (Fig. 6L); type **MC** – thick, with a truncated or slightly split apex (Fig. 6G). Microsetae: type **mA** – thin, pointed weakly sclerotized (Fig. 6J’); type **mB** – weakly sclerotized, cylindrical or broadened (Fig. 6I); type **mC** – strongly sclerotized and thick, with a blunt or truncated apex (Fig. 6H).

## Results

### Oviposition and duration of development of immature stages

Under laboratory conditions, two egg-laying periods were observed in *G. ruficollis*: one in the second half of May and the other in the first ten-day period of October. During the first period, the eggs laid in mud cells developed successfully, while in the second period all eggs were laid without mud cells and perished. Egg production correlated positively with the density of adult beetles in the cage. At a low density (one to three specimens of each sex), no egg-laying was observed. At an average density (four males and five females, or seven males and eight females), two eggs (0.4 eggs per female), and five eggs (0.62 eggs per female) were obtained, respectively. The main number of eggs (56, or 3.73 eggs per female) was received at a high density of specimens (13 males and 15 females).

The duration of the development from egg to imago amounted to 58–60 days. First instar larvae (mean = 10.59 days) and pupae (mean = 8.33 days) were the fastest to develop. The duration of the development of eggs and the second instar larvae was approximately the same (means = 14.35 and 13.22 days, respectively). The development of the third instar larvae took the longest time (mean = 19.0 days). Variations in the duration of the development of different stages are partly related to changes in biomass. During the development, the weight of eggs increased from 2.7 to 4.25 mg, that of the larvae of the first instar from 5.1 to 11.2 mg, the second instars from 13.1 to 22.7 mg, and the third instars from 26.4 to 102.8 mg, while the weight of the pupae changed insignificantly, from 101.2 to 103.6 mg. Thus, at the beginning of development, an approximately twofold increase in biomass is observed at each stage, and the feeding of the last instar larva provides an almost fourfold increase in body weight. Due to this, at the end of preimaginal development, the weight of a pupa almost reaches the weight of teneral adult (in average 109.5 mg) (Fig. 4).

### Descriptions of immature stages

#### Egg and mud cell (Figures 1, 2)

**Figure 1.**
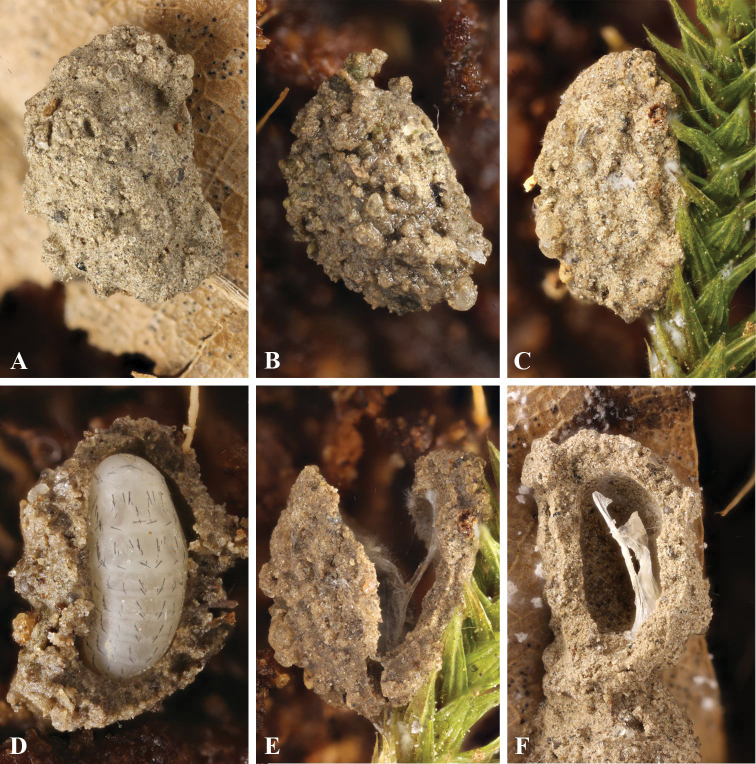
Mud cells of *G. ruficollis*: **A–C** mud cells on different substrates **D** egg in a mud cell **E, F** mud cells and remains of the chorion after larval hatching. Not to scale.

The egg is placed singly in the center of a mud cell (Fig. 1D) or loosely on the substrate. Mud cells are purse-shaped, fine-grained, and lined inside with fine grains of sand. The size (n = 17) is 4.7–5.2 mm (mean = 4.93) in length and 2.7–3.5 mm (mean = 3.14) in width. Females placed them one by one on various substrates: the lower surface of rotten leaves (Fig. 1A), on *Polytrichum* stems (Fig. 1C), but some were not attached to anything and placed on loose coconut chips (Fig. 1B).

**Figure 2.**
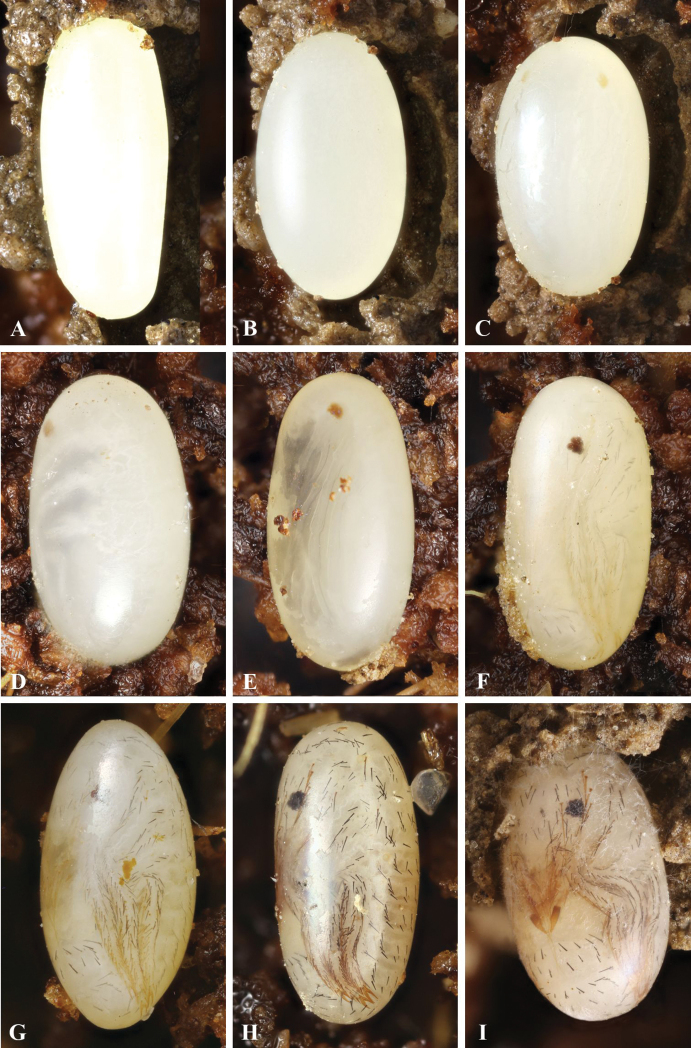
Eggs of *G. ruficollis* at different stages of the development: **A** immediately after oviposition **B** two days after oviposition, stage of the germ band **C** four days after oviposition, the appearance of eye spots and the beginning of leg differentiation **D** six days after oviposition, formation of the tracheal system **E** eight days after oviposition, full formation of legs and appendages **F–H** chaetotaxy developed after 9–10 days **I** larva just before hatching. Not to scale.

Immediately after laying the egg is white and oblong, 2.75–2.90× as long as width; the chorion is smooth, without distinct sculpture (Fig. 2A). During the development, egg proportions are changed noticeably: the length is slightly decreased, while the width is ca. 1.5× increased (Fig. 2D–G). On the last day of development, before the larva is hatched, the egg is only 1.65–1.75× as long as wide (Fig. 2H, I); superficially, the structure of the chorion fails to change. The size of an egg (n = 12) averages 2.46 × 1.48 mm.

#### First instar larva (Figures 3, 5A, 6, 7, 8B, D, 9, 10)

**Figure 3.**
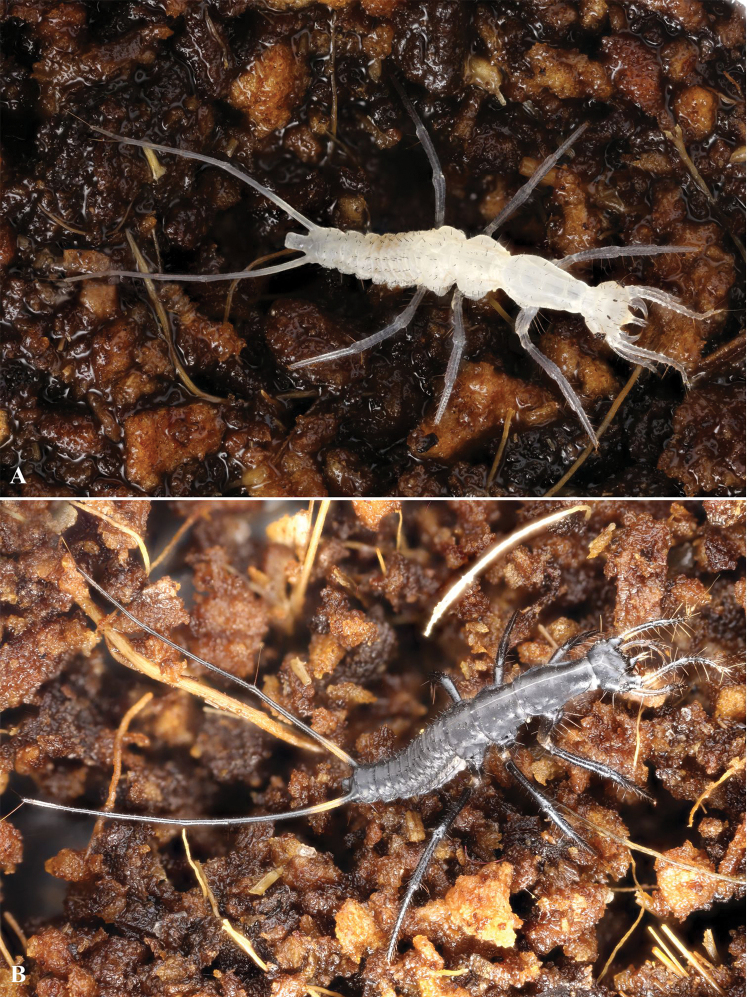
First instar larva of *G. ruficollis* after hatching **A** immediately after hatching **B** two hours after hatching. Not to scale.

***Habitus*.** Larva campodeiform, with a narrow body and very long appendages (Figs 3, 5A). Head small, with a narrow neck constriction (Fig. 7A–C), thorax unusually large (only 0.95–0.65× as long as abdomen). All three pairs of legs very long (Fig. 10A–C), apices of hind tarsi reaching the apex of abdomen (Fig. 5A). Urogomphi segmented, 1.5× as long as abdomen (Fig. 9L). Abdominal segment X short, cylindrical, slightly shorter than wide (Fig. 9K, L).

**Figure 4.**
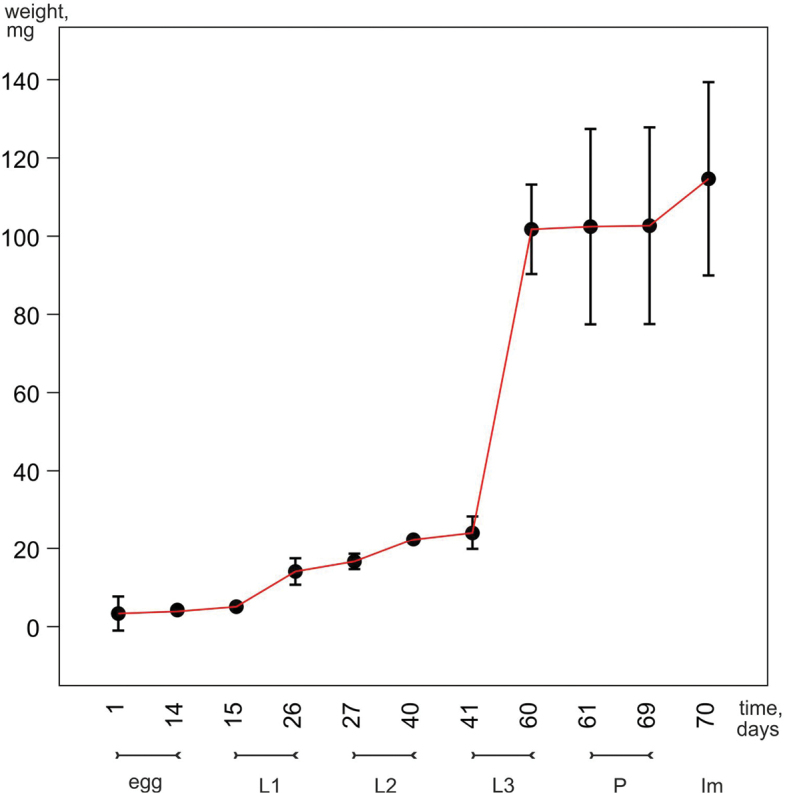
Time of development and change in mass (means and 95% confidence intervals) of preimaginal stages of *G. ruficollis*.

**Figure 5.**
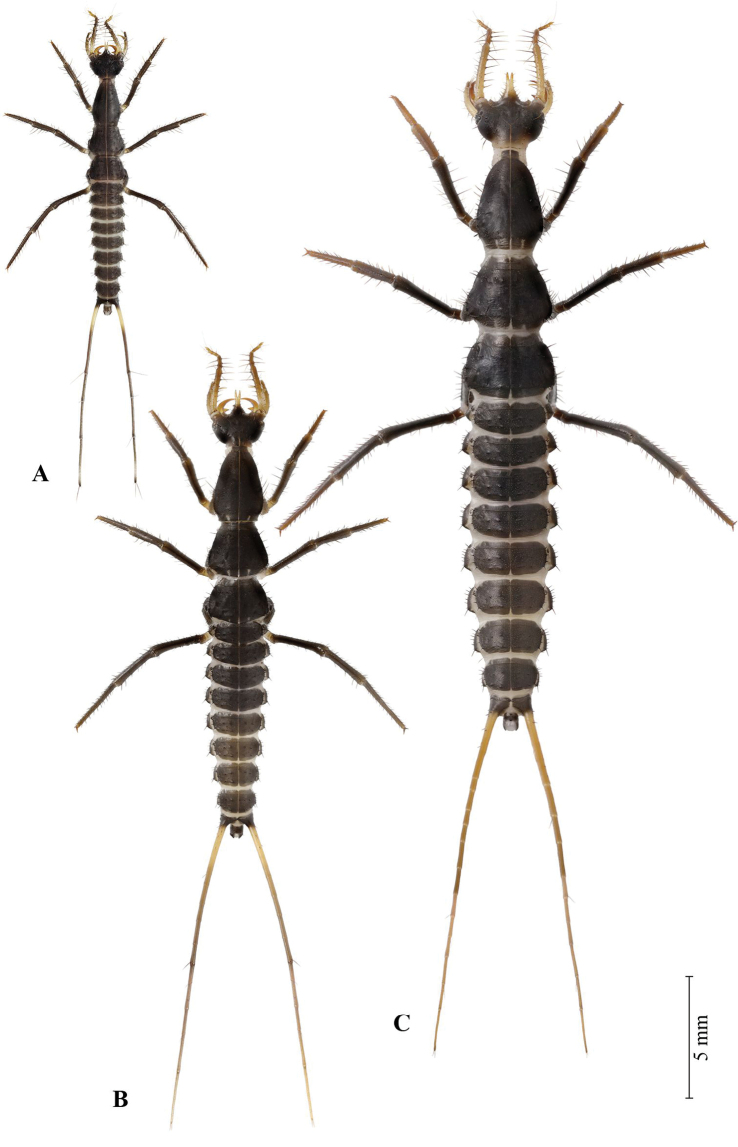
Habitus of *G. ruficollis* larvae **A** first instar **B** second instar **C** third instar.

***Body length*** (without urogomphi): 5.0–7.0 mm (mean = 5.9), urogomphi 5.7–8.6 mm (mean = 7.4) long.

**Figure 6.**
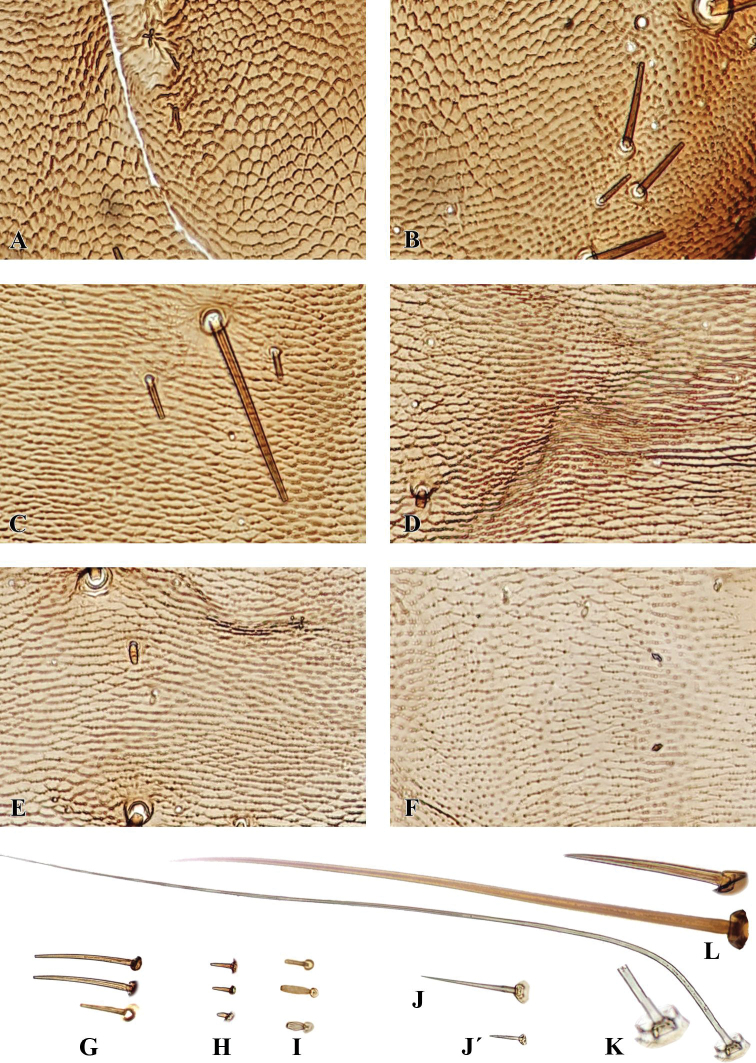
Microsculpture and the chaetotaxy of *G. ruficollis* larvae: **A–F** microsculpture (**A** frontale **B** parietale **C** prothorax **D** mesothorax **E** abdominal tergite **F** abdominal sternite) **G–L** types of chaetae (**G** MC **H** mC **I** mB **J** MA1 **J**’ mA **K** MA2 **L** MB). Not to scale.

***Color and sculpture*.** All sclerites strongly pigmented, most tergites almost black, pleurites and sternites brownish. Basal half of first antennomere, stipes, terminal antennomeres, mandibles, forehead outgrowth, trochanters, as well as basal portions and apices of urogomphi pale. Cuticle with a distinct microsculpture, this being mostly developed on sclerites; isodiametric on head (Fig. 6A, B) and transverse on tergites and ventrites (Fig. 6C–F); head appendages, legs and urogomphi with a rather weak transverse sculpture.

**Figure 7.**
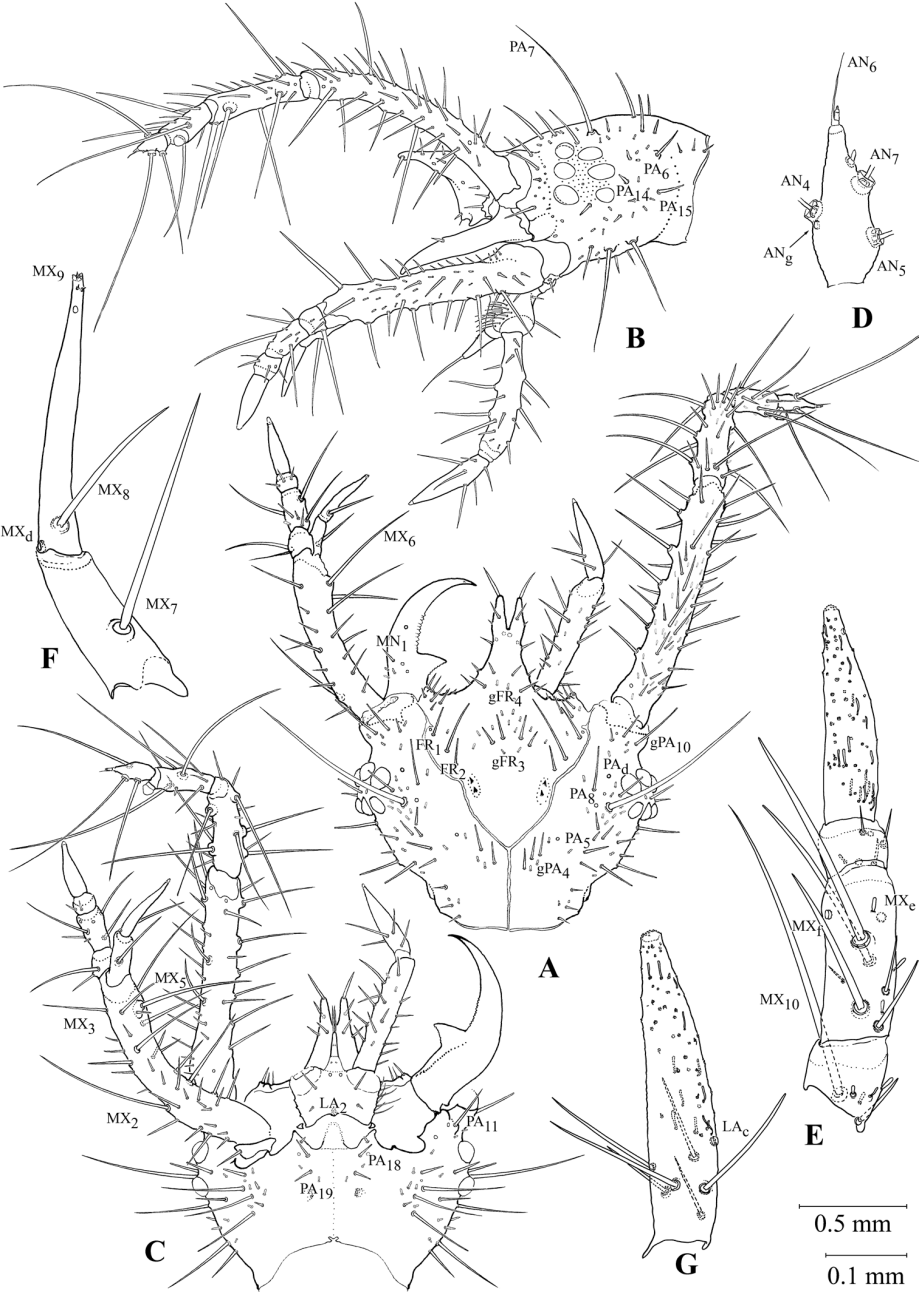
Head and the appendages of the first instar larva of *G. ruficollis***A** head, dorsal view **B** head, left lateral view **C** head, ventral view **D** apical antennomere **E** right maxillary palp **F** galea **G** apical segment of labial palp **A, C** with neither left antenna nor labial palp, nor right mandible. Scale bars: 0.5 mm (**A–C**); 0.1 mm (**D–G**).

**Figure 8.**
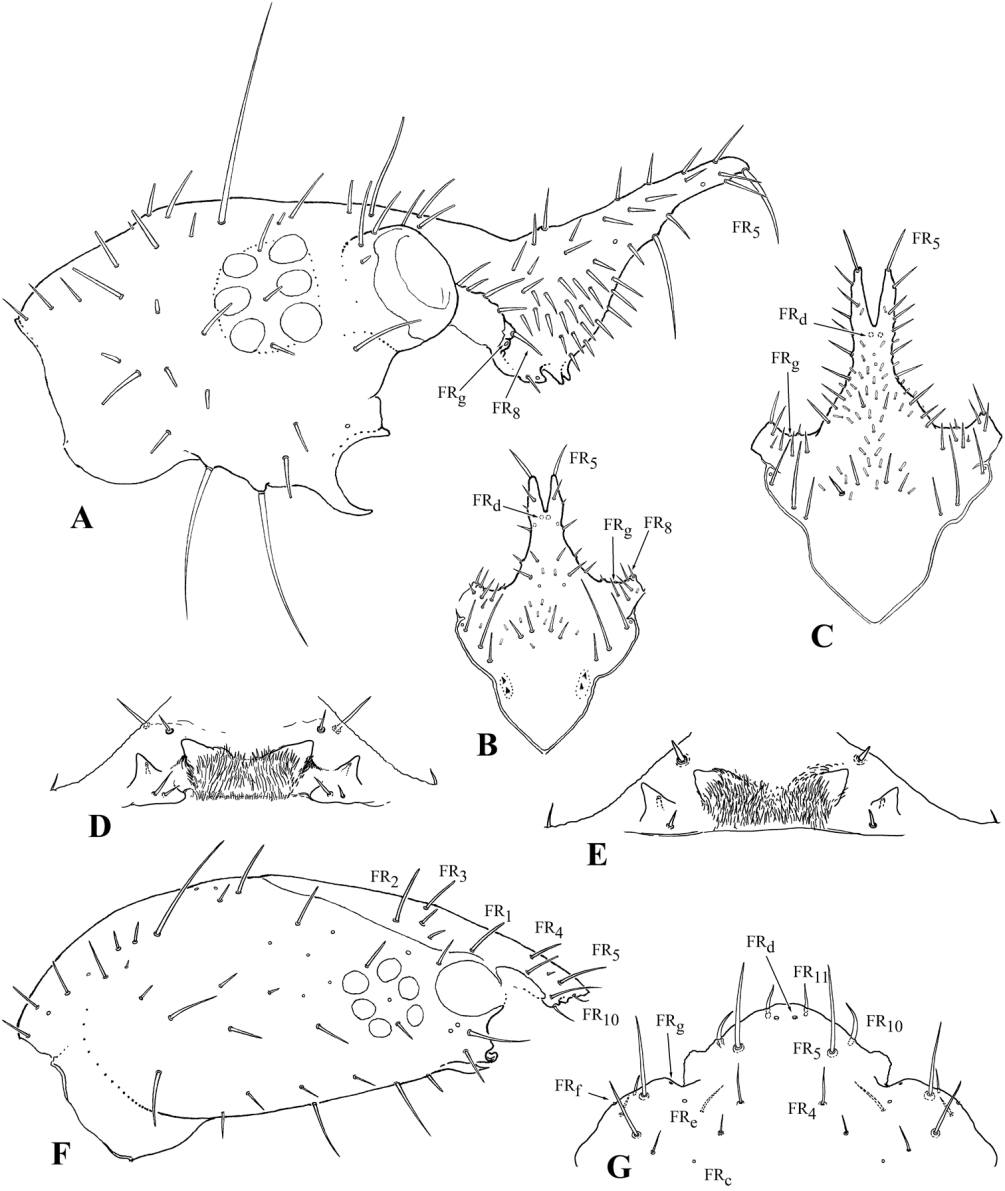
Head and nasale of larvae of the first (**B, D**), second (**C, E**) and third (**A, F, G**) instars **A–E***G. ruficollis***F–G***Drypta ussuriensis* Jedlička, 1963 **A, F** head, right lateral view **B, C** frontale, dorsal view **D, E, G** nasale **A** microsetae not shown. Not to scale.

***Head*.** Weakly transverse, 0.8–0.5 (mean = 0.7) × as long as wide, broadest near antennal rings, narrowing ca. 2.5× towards base, with a narrow neck-shaped constriction (Fig. 7A–C). Eyes large; eye tubercles large, slightly convex. Anterior part of frontal sclerite strongly drawn forward and forming a bifurcated protrusion (Figs 7A, 8B), the latter hanging over nasale (Fig. 8D). Outer angles of paraclypeus weakly protruding. Frontal sutures weakly curved, with two or three small egg teeth in area of sinuses. Postorbital and epicranial grooves absent. ***Antennae*** large, 3.5–4.0× as long as mandibles (Fig. 7A–C). First antennomere thick, slightly curved, half the total antennal length; second antennomere 0.25× as long as first and only slightly thinner. Antennomeres 3 and 4 much more slender, 3^rd^ antennomere asymmetrically articulated with apex of 2^nd^ and directed subrectangular both basal and lateral; terminal antennomere fusiform, with a narrow and pointed apex (Fig. 7D). ***Mandibles*** small, moderately curved, with a serrated distal part in front of a large triangular retinaculum, and a small undulate keel in basal part along ventral surface; penicillus missing (Fig. 7A). ***Maxilla*** ca. 0.85× as long as antenna (Fig. 7A–C). Cardo short, poorly sclerotized; stipes slightly curved, ca. 2/3 maxilla length; lacinia absent; galea 2-segmented (Fig. 7F), only slightly shorter than maxillary palps; apical joint narrow, conical, ca. 1.2× as long as basal one. Palps 4-segmented, 1^st^ and 3^rd^ segments very short, ca. 0.25× as long as other ones; apical segment pointed, with a small, rounded, sensory, apical area (Fig. 7E). ***Mentum*** trapezoid, transverse; ligula conical, large (longer than diameter of palpomere 1), strongly sclerotized, protruding far forward (Fig. 7C). Palps large, ca. 4× as long as mentum; 1^st^ palpomere almost 2/3 total palp length; 2^nd^ one fusiform, strongly narrowed towards apex, with a small, rounded, sensory area (Fig. 7G).

**Figure 9.**
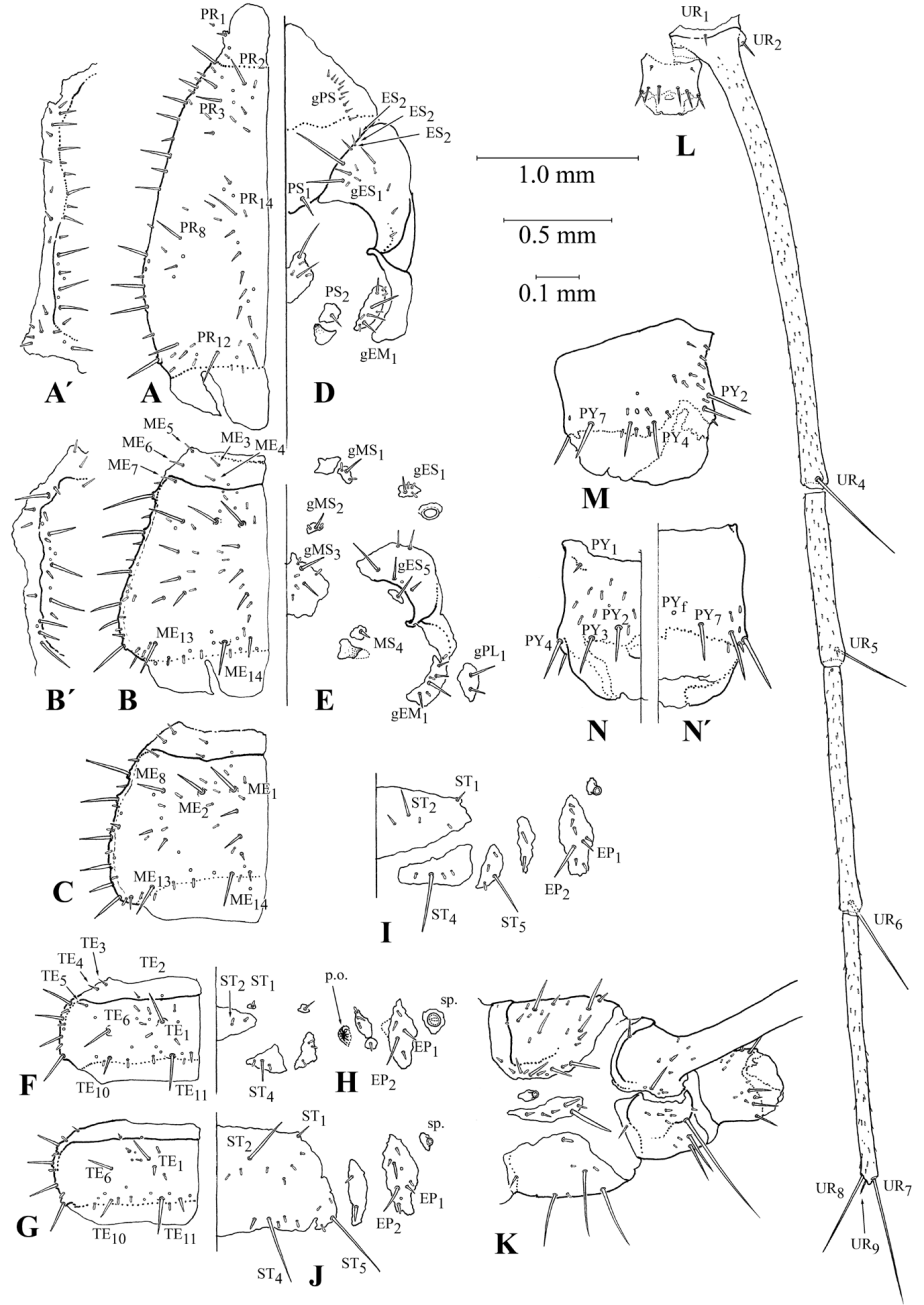
Body segments of the first instar larva of *G. ruficollis***A** pronotum, dorsal view (**A**’ its epipleura, lateral view) **B** mesonotum, dorsal view (**B**’ its epipleura, lateral view) **C** metanotum, dorsal view **D** sternites and pleurites of prothorax **E** sternites and pleurites of mesothorax **F** tergite of abdominal segment I **G** tergite of abdominal segment IV **H** sternites and pleurites of abdominal segment I **I** sternites and pleurites of abdominal segment III **J** sternites and pleurites of abdominal segment IV **K** abdominal segments VIII–X, lateral view **L** abdominal segments IX–X, dorsal view (left half of tergite IX not shown) **M, N** abdominal segment X (**M** lateral view **N** dorsal view **N**’ ventral view); p.o. – pleural organ or pleuropod; sp. – spiracle. Scale bars: 1.0 mm (**A–K**); 0.5 mm (**L**); 0.1 mm (**M, N**).

**Figure 10.**
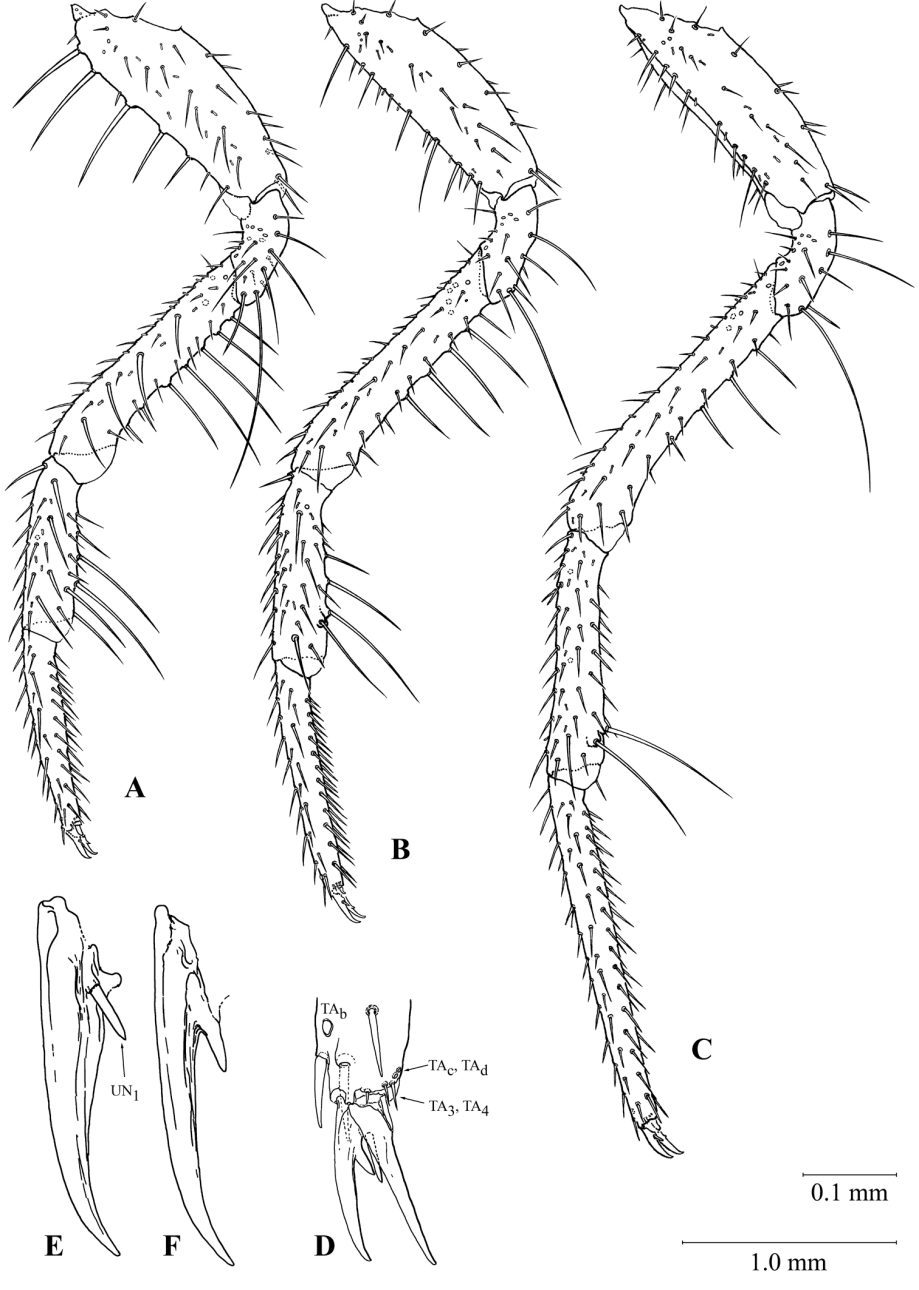
Legs of the first instar larva of *G. ruficollis***A, B, C** front, middle and hind legs, respectively **D** apex of tarsus, frontally and above **E, F** anterior and posterior claws, respectively. Scale bars: 1 mm (**F–C**); 0.1 mm (**D**); not to scale (**E, F**).

***Thorax*.** Tergites large, completely covering the dorsal surface of thoracic segments, with thickened and slightly curved lateral margins, without edging. Prothorax elongated, 2× longer than wide, strongly and conically narrowed anteriorly (Fig. 9A). Meso- and metasternum transverse, 1.2–1.5× wider than long, with distinctly thickened lateral margins (Fig. 9B, C); pretergite separated from tergite by a distinct and weakly curved carina. Area of sigilla of tergo-pleural and tergo-coxal muscles noticeably depressed. Sternites and pleurites rather strongly sclerotized. Not only a triangular prosternite, but also sclerotized medial areas well visible on prothorax (Fig. 9D). Small, irregularly shaped, additional sclerites located before base of furrows on all thoracic segments (Fig. 9D, E). Additional sclerites also developed in anterior portions of both meso- and metathorax (Fig. 9E). Epimere of prothorax divided into two parts by a membranous stripe; traces of this separation remaining on meso- and metathorax. Pleurites of both meso- and metathorax small, strongly pigmented (Fig. 9D, E). Trochantin small, oval. ***Legs.*** Long, rather slender, especially hind pair (Fig. 10A–C). All coxae subequal in length, subcylindrical, slightly narrowed distally. Trochanter 0.25× as long as coxa along dorsal surface. Distal sections, especially tibia and tarsus, highly elongated and clearly different in different pairs: fore tarsi shorter than tibiae; middle tarsi and tibiae virtually same in length, while hind tarsi slightly longer than tibiae. Tarsi with two claws virtually the same in size, but different in structure (Fig. 10D): anterior claw with a large, basal, spiniform seta (Fig. 10E), while posterior claw with a basal outgrowth similar in size and shape, but without seta (Fig. 10F).

***Abdomen*.** Tergites strongly sclerotized and pigmented, transverse, 1.7–2.7× longer than wide, with a strong carina slightly projecting laterally between pretergite and tergite (Fig. 9F, G). Spiracles rather large, especially so in segment I, strongly and conically protruding beyond body contours (Fig. 9H). Epi- and hypopleurites distinctly sclerotized, hypopleurite always short and narrow (Fig. 9H–J). Abdominal segment I with a developed pleural organ or pleuropod (Fig. 9H). Degrees of consolidation of ventrites differing markedly in different abdominal segments. Both pre- and poststernites in abdominal segments I and II separated from mesosternite (Fig. 9H); presternite in segment III connected with mesosternite (Fig. 9I); starting with segment IV, all sternites forming a common ventral sclerite (Fig. 9J); hypopleurites in segment VIII included into ventral sclerite (Fig. 9K), while a unified complex ventrite formed in segment IX (Fig. 9K, L). ***Urogomphi*.** Long, fused fast with tergite IX; their distal parts consisting of three segments separated by membranous annular folds (Fig. 9L). Tergite IX short, divided by a medial membranous stripe interrupted by a peculiar joint substituting an anterior carina. Tergite IX thus represented by two movably connected halves.

***Chaetotaxy*.** Frontale showing all basic elements, but unusual in their topology (Figs 7A, 8B). In basal portion, positions of setae similar to typical: FR_b_ as well as FR_1_, and FR_2_ (both MC) located near outer branches of frontal sutures; gFR_3_ (usually two pairs of MC) in central part of forehead. gFR_6-7_ including four MB and a small lateral seta at outer angles of paraclypeus, being relatively easy to identify. FR_8_ terminal in position at anterior angles of paraclypeus (Fig. 8B), being easily identifiable due to a medially placed FR_g_ located on a tubercle under FR_8_ and, thus, hardly visible even on slides. Location of other sensilla on frontale strongly distorted by a long bifurcate outgrowth, which in most previous publications was designated as a nasale (see Discussion). In this case, the identification of groups of sensilla is only possible using additional criteria: the presence of sigilla and the stable combinations of different types of sensilla. The sigilla of the cybarium dilator clearly separate gFR3 from gFR4, including three or four pairs of MB and MC, being located at the base of the outgrowth. gFR_5_ occupies the dorsal surface of the outgrowth, while a pair of thick, curved, apical macrosetae is to be considered as actually representing FR_5_, because large sensilla located ventrally and laterally are very likely to be homologs to FR_d_ and FR_e_. A small seta located between FR_g_ and the base of the outgrowth at the anterior margin of the frontale can be interpreted as a medially displaced FR_9_. The nasale is placed ventrally at the base of the outgrowth and consists of lateral teeth and a two-lobed medial protrusion (Fig. 8D). Lateral teeth with a subapical conical sensilla FR_11_ and a laterobasal FR_10_ (mA). Thus, among the primary sensilla of the frontale, only the presence and/or position of FR_c_ remain unclear yet. In addition, two or three dozen not always paired mB are present on the frontale.

Parietale with numerous secondary setae of various types (Fig. 7B, C). gPA_10_ (three MC differing in length) placed on antennal rings. PA_4_, gPA_5_, PA_6_, PA_8_, PA_9_, PA_11_, and PA_13_ represented by similar macrosetae (MC). PA_1_–PA_3_ (MA1) located in occipital area. gPA_14_–gPA_15_ and gPA_16_–PA_17_, each with a pair of very large MB and one or two smaller setae occupying the ventrolateral surface. Setae of gPA_16_–PA_17_ borne on small tubercles. In the gular area, the generalized pool of setae and sensilla, like PA_18_, PA_19_, and PA_o_, is preserved. PA_b_ is replaced by MC. Like in the frontale, two or three dozen mB are located there.

Antennae showing a highly complicated and differentiated chaetotaxy pattern (Fig. 7A–C). Except for the typical AN_a_–AN_e_, the 1^st^ antennomere is clothed with numerous setae. Its dorsal and inner surfaces, especially in the distal part, are with large MB, the bases of the largest ones being located on tubercles. In addition, this antennomere has at least 15 MC and numerous mB. The 2^nd^ antennomere is with eight MC located on tubercles, ten mC, and several mB. The longest and thickest setae on those antennomeres are mainly directed basad and mesad, resembling a trapping apparatus. Besides setae, the 3^rd^ antennomere is with AN_f_ and a small, conical, sensory appendage with one bell-shaped and two short (2–3× shorter than sensory appendage) conical sensilla at the base. The 4^th^ antennomere has the basic set of sensilla: AN_g_, very long and thin AN_4_, AN_5_, AN_7_ (MA2), two conical sensilla (one distal to AN_5_, the other at apex), and terminal microseta AN_6_ (Fig. 7D). Apical and preapical antennomeres are specialized as sensory. Their macrosetae are very long and thin, protruding far beyond the apices of a few mC and mB.

Mandibles with all primary setae (MN_1_, MN_2_) and sensilla (MN_a-c_); moreover, MN_b_ doubled, and outer edge in front of base with a group of four or five mB (Fig. 7A).

Cardo with a single MX_1_ (MC), stipes without separate gMX along inner margin, its chaetotaxy resembling that of the 1^st^ antennomere: seven or eight very large MB, including MX_2_, MX_3_, MX_4_, MX_5_, located on tubercles; two dozen MC and several dozen mB (Fig. 7A–C). Lacinia replaced by a small tubercle with MX_6_ (MB) and mC. Galea without secondary sensilla, with MX_7_ and MX_8_ (MB) at least half galea length and borne on small tubercles. MX_d_ located typically at the base of the 2^nd^ galeomere; apical part of the 2^nd^ galeomere with one or two subapical conical sensilla gMX_9_, one placoid sensilla, and an apical sensory area with one conical and four bell-shaped sensilla (Fig. 7F). The 1^st^ palpifer with a very large MX_10_ (MB) and four or five mB; the 2^nd^ palpifer, in addition to the usual MX_e_ and MX_f_, bears three large MB, three or four MC of a significantly smaller size, and four or five mC; the 3^rd^ palpifer shows a practically basic set of setae and sensilla: in addition to small MX_11_, MX_12_ (MA1) and MX_g_, it has one or two mC and small mB. Apical segment of maxillary palpus with subapical placoid sensilla, as well as numerous conical and finger-shaped sensilla, all rather evenly spaced; only finger-shaped sensilla somewhat denser laterobasally (Fig. 7E).

Chaetotaxy of ventral surface of submentum with typical setae LA_1_ and LA_2_, as well as sensilla LA_a_ (Fig. 7C). Prementum laterally and dorso-apically with one or two rows of thin, flexible, long setae gLA_3_–gLA_4_–gLA_5_. Apex of ligula with LA_6_ (MB) and poorly distinguishable, often asymmetrically located LA_7_ (mA). The 1^st^ segment of labial palpus, except placoid sensilla LA_b_, with 16–20 MB and MC, as well as 10–12 mB. The 2^nd^ segment of labial palpus in basal part with a circular group of six MB, a distally located LA_c_, as well as rather evenly spaced conical and finger-shaped sensilla (Fig. 7G).

Pronotum with numerous additional setae of various types. Only PR_1_ is typical, while the other setae are represented by MC, mB, and mC. The number and arrangement of mB is highly variable even between specimens. Therefore, only the arrangement of more constant MC and mC is described below. gPR_2_ and gPR_3_ (one MC and two mC in each group) are located along the anterior margin of the pronotum, while gPR_14_ (one MC and 8–10 mC) extended to posterior margin, as well as gPR_8_ (one MC and one or two mC) placed on the disc (Fig. 9A). gPR_6_ with four or five MC and the same number of mC is located in anterior half of lateral margin. Probably PR_4_ and PR_5_ are also to be included in this group, but they are indistinguishable among secondary setae; gPR_9_–gPR_11_ are organized similarly. Posterior margin of pronotum with gPR_13_–PR_14_ (two MC and two mC). Pronotal epipleura with a small group of 10–12 secondary setae posteriorly (Fig. 9A’). Prosternite with a typical set of setae: single PS_1_ and gPA (ca. ten mA) on anterior membrane. Other ventrites and pleurites with additional setae. A small sclerite, at the base of furca, with PS_2_, and the medial secondary sclerite with two pairs of MC, and three pairs of mB. Episternum with gES_1_ (two MC and five mC), and epimere with gEM_1_ (one MC and three mC) (Fig. 9D).

The chaetotaxy of the prothorax in *Galerita* larvae is an interesting example of serial homology. Based both on the location of sclerites in relation to the endoskeleton (notum and furca) and on the places of muscle attachment in different thoracic segments, PS_2_ corresponds to MS_4_, while MC of the medial secondary sclerite of the prothorax corresponds to MS_2_ and MS_3_. It is noteworthy that in most larvae of ground beetles these setae are entirely absent.

The chaetotaxy of the meso- and metathorax is organized similarly. Meso- and metanotum with typical small ME_3_, ME_4_, ME_5_, ME_6_, ME_7_, and ME_a_ (Fig. 9B). Other setae forming complex groups: gME_1_ with two or three MC and one or two mC; gME_8_ with one MC and one–three mC (Fig. 9C); laterally located gME_9_–ME_12_ with six MC and five or six mC. Secondary setae on sternites represented mainly by mC, and only gMS_3_ with two MC. Chaetotaxy of pleurites organized in the same way; only gPL_1_ (only on mesothorax) and gES_5_ with additional MC (Fig. 9E). A short row with two or three mA in the anterior part of the episterna in all thoracic segments is an interesting feature of the chaetotaxy of *G. ruficollis*.

Legs with particularly complex and differentiated setae (Fig. 10A–C). As the other appendages, legs with many mC grouped with MB, but not with MC. Segments of legs with an entire primary set of setae and sensilla, except UN_2_. In most cases, additional setae form distinct rows, especially on the ventral surface. On femur and tibia, MB and mC alternate evenly in these rows; on tarsus, these rows are rather homogeneous. The differentiation of MC differentially expressed on the fore-, middle and hind legs is the most specific feature of their chaetotaxy. Fore coxae with five tubercles on anterior rib, bearing very thick and slightly curved setae (Fig. 10A); middle coxae without tubercles, but with shorter setae reduced in number (Fig. 10B); fore coxae entirely without such setae (Fig. 10C). Fore femora with two rows of tubercles and each bearing five thick curved setae at anterior and lower margins (Fig. 10A); on middle femora such five specialized setae placed only on ventral surface (Fig. 10B); hind femora with only three small setae in ventral row (Fig. 10C). Fore tibiae with two ventrolateral rows of three or four setae (Fig. 10A); middle tibia with only one, distal seta in anterior ventrolateral row (Fig. 10B); hind tibia with only two subapical setae (Fig. 10C). Thus, the differentiation of the chaetotaxy of the legs, especially forelegs, is similar to that of the antennae and maxilla. Dorsal surface of the tarsus with well-developed TA_a_ (subapical) and TA_b_ (at the border of basal third of the tarsus), TA_1_ absent or indistinguishable from surrounding secondary setae. The set of apical tarsal setae and sensilla is arranged in a peculiar way. It includes usual and very constant in Carabidae TA_3_, TA_4_, TA_5_, TA_6_, and TE_c_, TA_d_, TA_e_, TA_f_ (Fig. 10D). In addition, a peculiar conical sensilla is located above TA_3_; UN_2_ absent, while UN_1_ thick and spiniform, placed at the base of the anterior claw (Fig. 10E).

Abdominal tergites are also with a complex chaetotaxy pattern which is formed, besides sensilla, also by setae MC, mB and mC. TE_2_, and TE_3_ (abdominal segment I also with TE_4_ and TE_5_) on pretergite very small and rather thin (Fig. 9F). Large TE_1_, TE_6_, TE_10_, and TE_11_ on tergal discs surrounded by additional mB and mC, as well as sensilla of other types (Fig. 9F, G). gE7 and gTE9 (each with two MC and two or three mC) are located laterally. Epipleurites, in addition to rather large EP_1_ and EP_2_ (MC), usually with 4–7 mC and 2–4 mB. Hypopleurites usually with two or three mC and one or two mB (Fig. 9H–J). All sternites with ST_1_ (on sternites I and II located on a separate pretergite), ST_2_, ST_4_, and ST_5_ (all MC); tergites I–III without ST_3_, on the other ones this seta is present, sometimes only on one side; ST_6_ missing; besides theses setae, sternites with numerous additional mB (Fig. 9H–J).

Urogomphi with an almost typical set of macrosetae: smaller UR_1_ at border of pretergite, and larger UR_2_ at posterior angles of tergite IX. Each articulation in distal part of urogomphi located at base of corresponding macroseta: dorsal UR_4_, lateroventral UR_5_, and ventral UR_6_. Apex of urogomphi with a typical set of two macrosetae UR_7_ and UR_8_, and microseta UR_9_. Surface of urogomphi rather uniformly clothed with numerous (more than two hundred) mB and even more small sensilla of other types, mainly numerous, elongated, bell-shaped sensilla, resembling TR_b_–TR_f_ cuticle stretch receptors. Against the background of these numerous structures, no correct identification of UR_a_–UR_g_ sensilla is possible (Fig. 9L).

Due to the shortening of segment X, most of homologous setae (except PY_1_) are displaced distally and form a ring with PY_2_ and PY_3_ in the dorsal part, PY_4_–PY_6_ in the lateral part, and PY_7_ in the ventral part. Ten to twelve pairs of mB are located mainly on the dorsal surface of the pygidium, and only a few such setae are placed on its lateral surface (Fig. 9M, N).

#### Second and third instar larvae (Figures 5A, C, E)

Larvae of the older instars differ in color: their head appendages and urogomphi are entirely light, the fore tibiae and fore tarsi are noticeably lightened, especially in the third instar larva (Fig. 5B, C). With each molt, the size of the larvae is increased. The body length in the second instar larva is 7–9 mm, in the third instar it is 11–13 mm; the urogomphi length is 10.4–11.3 mm and 14.6–16.6 mm, respectively. Moreover, in the older larvae, the number of urogomphi segments is increased as well: 10–12 (mean = 11) in the second instar, and 12 or 13 in the third instar. Additionally, the number of secondary setae increases. Thereby an increase in the number of secondary setae in the anterior part of the head and on its appendages, on the outgrowth of the forehead (Fig. 8C vs. Fig. 8B), as well as on the abdominal pleurites and ventrites is especially noticeable. On the contrary, in the basal part of the head, on the legs, and on the abdominal tergites the number of secondary setae is increased only insignificantly.

### Diagnosis of the larvae

Generally, the larva of *G. ruficollis* is similar to the previously described larvae of other *Galerita* species. It differs from all known larvae of *Galerita* by the structure of the claws, of which the posterior one has a large tooth, dark head and tergites, as well as yellow mouth appendages and urogomphi. The data available from the previous descriptions do not allow us to include the features of chaetotaxy in the diagnosis. It seems possible that sensilla FR_b_ and PA_b_ are replaced by setae.

#### Pupa (Figures 11, 12A)

**Figure 11.**
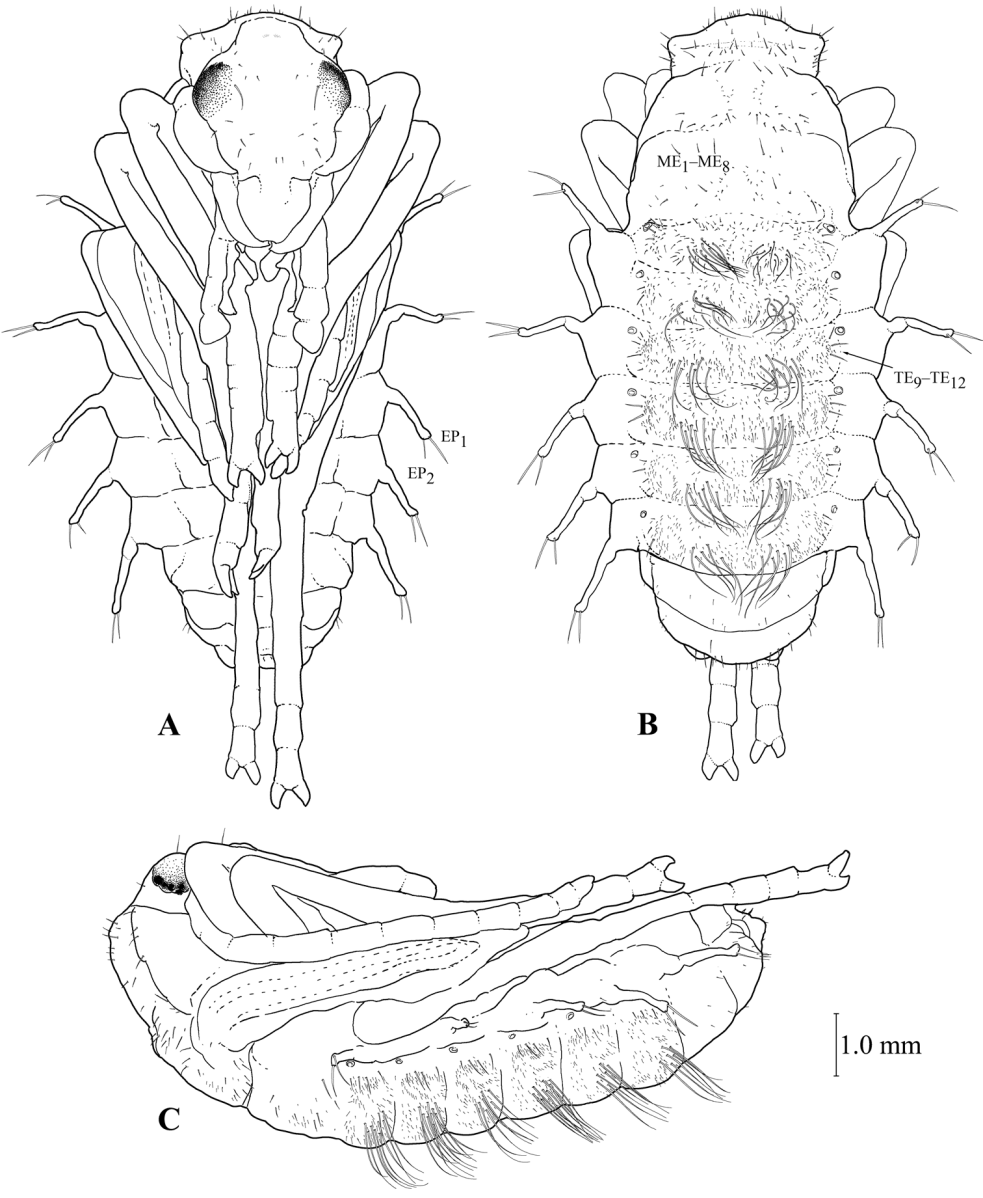
Pupa of *G. ruficollis***A** ventral view **B** dorsal view **C** left lateral view.

The pupa has a structure typical of all ground beetles. The proportions of the appendages and the shape of the head are generally like in the *Galerita* adult. Labium with a slight notch at apex. Pleurites II–VI with long, thickened, apical outgrowths. Wings, legs, and head appendages without setae, while thoracic and abdominal segments with a peculiar chaetotaxy pattern. Tergites I–VI of abdominal segments with paired groups of long and strong setae, on which the pupa lies on the substrate. In addition, these tergites are covered with numerous microsetae, and their lateral sides bear thicker setae which topologically correspond to gTE_9_–gTE_12_ of the larva. Pleural outgrowths with a pair of large chaetae, these probably corresponding to larval EP_1_ and EP_2_ (Fig. 11A). Abdominal tergites VII and VIII without microsetae, but with only few marginal and medial setae. Meso- and metanotum with groups of microsetae at base and lateral to the midline, as well as with well-defined anterior and posterior rows of setae, these topologically corresponding to ME_1_–ME_8_ and ME_13_–ME_14_ (Fig. 11B).

**Figure 12.**
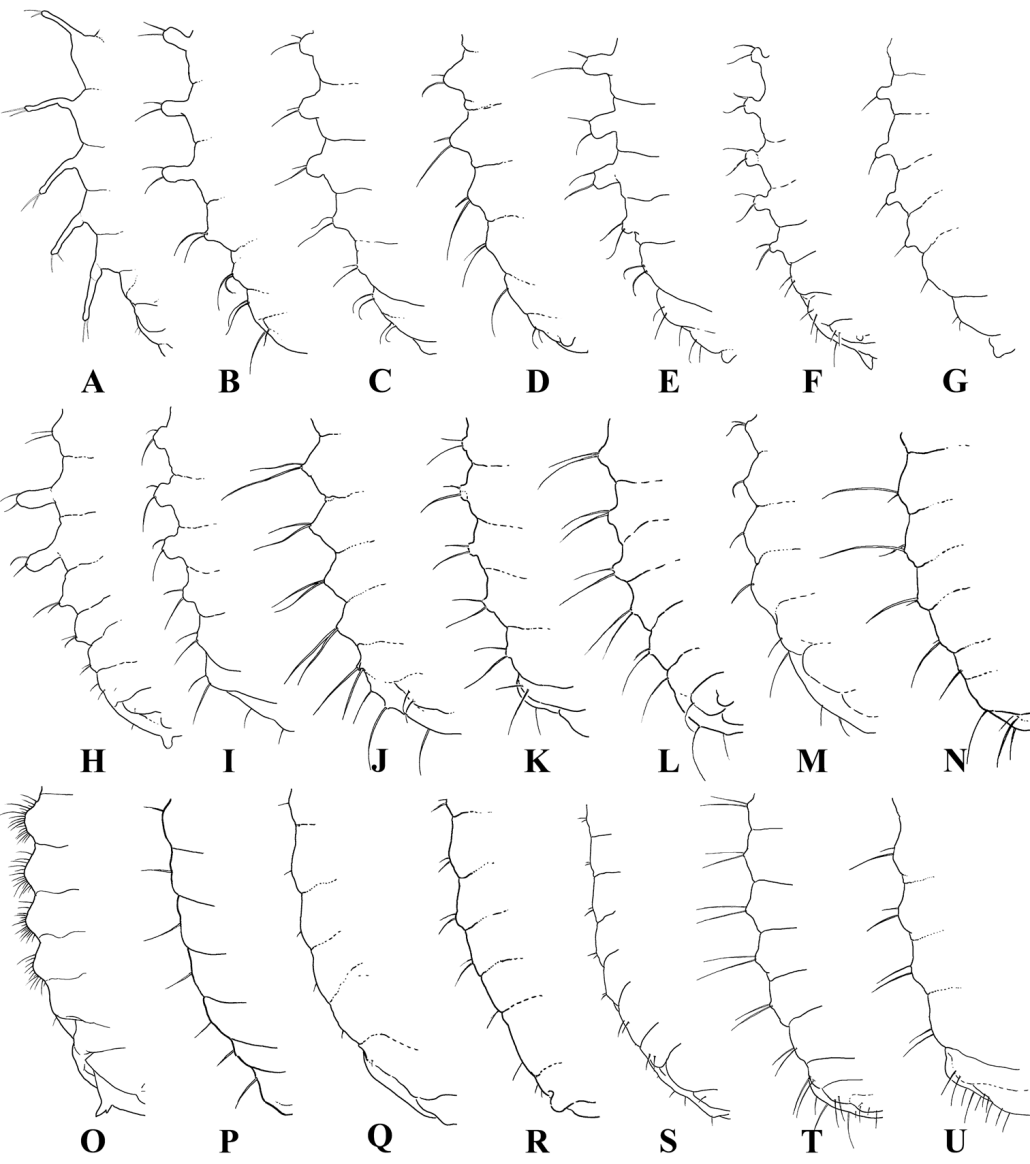
Pleural areas of pupae of ground beetles, ventral view **A**Galeritini (*G. ruficollis*) **B–D**Platynini (**B***Metacolpodes buchanani* (Hope. 1831) **C***Limodromus assimilis* (Paykull, 1790) **D**Agonum (Olisares) sculptipes (Bates, 1883) **E–G**Pterostichini (**E**Pterostichus (Pseudomaseus) nigrita (Paykull, 1790) **F**Pterostichus (Lenapterus) costatus (Ménétriés, 1851) **G**Poecilus (Poecilus) reflexicollis Gebler, 1830) **H**Chlaeniini (Chlaenius (Achlaenius) variicornis A. Morawitz, 1863) **I**Oodini (Oodes (Oodes) integer Semenov, 1889) **J**Licinini (Diplocheila (Isorembus) minima Jedlička, 1931) **K** – Odacanthini (Odacantha (Odacantha) puziloi Solsky, 1875) **L–N**Harpalini (**L**Stenolophus (Stenolophus) propinquus A. Morawitz, 1862 **M**Harpalus (Pseudoophonus) sinicus Hope, 1845 **N**Dicheirotrichus (Trichocellus) placidus (Gyllenhal, 1827) **O**Carabini (Carabus (Coptolabrus) smaragdinus Fischer von Waldheim, 1823) **P**Pogonini (Pogonus (Pogonus) transfuga Chaudoir, 1871) **Q–S**Zabrini (**Q***Harpalodema fausti* Reitter, 1888 **R**Amara (Celia) saginata (Ménétriés, 1847) **S**Amara (Curtonotus) alpina (Paykull, 1790)) **T, U**Lebiini (**T***Parena tripunctata* (Bates, 1873) **U**Cymindis (Tarsostinus) lateralis Fischer von Waldheim, 1820). Not to scale.

## Discussion

### Seasonal activity of the immature stages

At present, only brief information about the seasonal activity of the immature stages of Galeritini species is available. In the Northern Hemisphere the third instar larvae and the pupae of four *Galerita* species (*G. janus* (Fabricius, 1792), *G. lecontei* Dejean, 1831, *G. nigra* Chevrolat, 1835 and *G. simplex* Chaudoir, 1852) were collected in U.S.A. and Mexico from July to October, mostly in late July to early August (Sallé 1846; Candèze 1861; Hubbard 1875; Schaupp 1878b). On the contrary, in the Southern Hemisphere the larvae and pupae of *Galerita carbonaria* Mannerheim, 1837 and *Galerita brasiliense* Dejean, 1826 were found in Brazil from October to February (Costa et al. 1988), while the third instar larvae of *Trichognathus marginipennis* Latreille, 1829 were collected in Paraguay in November and April (Arndt and Drechsel 1998), but in Brazil they were found in late July (dos Santos 2012).

Generally, our data correspond well to the results of the previous observations. Because the oviposition period for successfully developing eggs of *G. ruficollis* started in the second half of May, the appearance of pupae and the emergence of the new generation of adults were naturally observed in early to mid-July. Similar periods of reproduction and emerging of some *Galerita* species were observed in USA. For example, the copulation of *G. bicolor* in Indiana is observed in May (Larochelle and Larivière 2003), the activity of teneral beetles of *G. janus* in New York is started in August (Larochelle and Larivière 2003), while in Tennessee the activity of the second and the third instar larvae of *G. janus* is observed in mid-August, pupation took place in the last ten-days period of August and the emerging of teneral adults is recorded in end of August – start of September (Dajoz 2005). Considering the inversion of the year’s seasons in the Northern and Southern Hemispheres, according to Matalin (2007), Galeritini species can be characterized as spring-summer breeders.

### Oviposition technique and the peculiar development of immature stages

The first information concerning the unusual egg-laying technique in Carabidae which implies the creation of mud cells was noted by Bargagli (1874) for the European *Percus passerini* (Dejean, 1828). Later, Riley (1886) also found the eggs of the American *Chlaenius impunctifrons* Say, 1823 in mud cells. He suggested that this technique was also typical of *Chlaenius aestivus* Say, 1823 and *Scarites subterraneus* Fabricius, 1775, as well as the species of the genera *Dicaelus* Bonelli, 1813 and *Galerita* Fabricius, 1801. Mud cells of four *Chlaenius* Bonelli, 1810 species (*C. aestivus*, *C. impunctifrons*, *C. sericeus* Forster, 1772, and *C. tricolor* Dejean, 1826), as well as *Brachinus cyanipennis* Say, 1823 and *Galerita bicolor* Drury, 1773 were described and illustrated for the first time by King (1919). Until now, this description of mud cells of *Galerita* has remained the only one. According to the available information, the shape and size of mud cells in both *G. bicolor* (King 1919: fig. 3) and *G. ruficollis* (Fig. 1) are very similar. Purse-shaped mud cells can well be assumed as being characteristic of *Galerita* species.

It seems noteworthy that a gregarious behavior during cooperative hunting of the adults and larvae has been recorded in *Trichognathus marginipennis* (dos Santos 2012). Moreover, this species shares the same environment and forms of aggregation with species of *Tetracha* Hope, 1838 (Arndt and Drechsel 1998; dos Santos 2012). Besides Galeritini, multispecies aggregations of different species of *Brachinus* Weber, 1801 (Wautier 1971; Juliano 1985; Schaller et al. 2018), as well as aggregations of some *Brachinus* species with *Anchomenus dorsalis* (Pontoppidan, 1763) and/or with *Chlaenius chrysocephalus* (P. Rossi, 1790) have also been observed (Zaballos 1985; Bonacci et al. 2004, 2011; Zetto-Brandmayr et al. 2006). Both gregariousness and aposematic coloration are considered to increasing both the effect of the aposematic signal on predators and the level of chemical protection (Bonacci et al. 2011; Schaller et al. 2018). However, virtually nothing is known about the influence of aggregation (group effect) on the egg-laying process not only in Galeritini, but in Carabidae as a whole. Only Schaupp (1878b) found one larva of *G. janus* in a cage containing two males and four females. We obtained similar results, because at the average density of adults in the cage, the egg production in females was minimal.

At last, data on the duration of the development of any immature stages of Galeritini is very scant. Only the duration development of the pupae of *G. carbonaria* (Costa et al. 1988) and *G. janus* (Dajoz, 2005) is known and it is ca. 6–8 days, that is well corresponded with our data. According to the data obtained in laboratory conditions, the development of *G. ruficollis* from egg to adult takes ca. two months, and the presence of two periods of oviposition suggests two generations per season. In this case, the development of the eggs laid in the first half of October is to be completed in the first half of December. In the tropical belt, where the bulk of the distribution area of *G. ruficollis* is located (Reichard 1967), this is theoretically possible. However, in autumn, during the next oviposition, the females did not place the eggs in mud cells but laid them freely on the substrate. Moreover, after laying the eggs in one section of the cage, after some time the females built empty mud cells in another section of the same cage. As it was mentioned above, not a single egg managed to develop without mud cells. This can be taken as an indirect evidence of two generations being impossible for this species to realize during one season.

### Comparison of Galeritini immature stages

All previous authors considered the bifurcated protrusion at the anterior margin of the frontale (Figs 7A, 8A–C) as a “nasale” (van Emden 1942; Thompson 1979; Costa et al. 1988; Arndt and Drechsel 1998; Dajoz 2005; Bousquet 2010; dos Santos 2012). Moreover, none of them, except Costa et al. (1988), paid attention to the paired teeth lying ventrally at its base (Fig. 8D, E). However, the correspondence of this protrusion to the real nasale in the larvae of other Carabidae has long raised obvious doubts. Firstly, a real nasale can strongly vary in structure and tooth armament, but two pairs of setae, FR_10_ and FR_11_, are its constant feature. Secondly, in the process of larval growth, the shape, size, and number of teeth of the nasale can change significantly, but secondary setae are never to be formed in this case. The paired protrusion on the head in the larvae of *Galerita* supports numerous setae of different types, and their number is increased with each molt, just as it takes place over the entire frontale. Based on the features of chaetotaxy, Makarov (2002) has long established the homology of this structure with the anterior part of the frontale, i.e., anterior to FR_3_. After the study of *Galerita* larvae of different instars, as well as a serial material of the larvae of Dryptini, this homology has become fully clarified. In the larvae of *Drypta* Latreille, 1796, similar trends are observed, albeit to a lesser extent. Both FR_5_ and FR_d_ are moved forward and situated far in front of FR_8_, while FR_g_ occupy a relatively medial position (Fig. 8F, G). Thus, we can clearly identify all frontal setae in *Galerita*, except FR_10_ and FR_11_. The localization area of both latter setae is devoid of additional markers and, at the same time, is strongly transformed. Due to the presence of numerous secondary setae, two options can be considered: either FR_10_ and FR_11_ have retained their original positions near the teeth of the nasale or they could have shifted to the ventrobasal part of the frontal protrusion.

The pupae of Galeritini differ from those of all other Carabidae in two characteristic features: long pleural outgrowths (Figs 11A, B, 12A) and numerous microsetae on abdominal tergites (Fig. 11B, C). Such setae are not known in the pupae of other ground beetles, even if the larvae have numerous microsetae on the tergites like Chlaeniini and some Lebiini. Unfortunately, since this important detail has not been noted in the available descriptions of pupae of all other *Galerita* species (Sallé 1846; Schaupp 1882; Costa et al. 1988; Dajoz 2005) as well as in *T. marginipennis* (dos Santos 2012), we are unable to assess the significance of this trait yet. On the contrary, long outgrowths on abdominal segments II–VI (Fig. 11A, B) have been described in all pupae of Galeritini (Sallé 1846; Schaupp 1882; Costa et al. 1988; dos Santos 2012). Based on their topology and chaetotaxy, these outgrowths correspond to larval epipleurites (Fig. 11A). Such outgrowths, albeit smaller ones, are known in the pupae of Platynini (Fig. 12B–D), Pterostichini (Fig. 12E–G), Chlaeniini (Fig. 12H), Oodini (Fig. 12I), Odacanthini (Fig. 12K), some Harpalini (Fig. 12L) (our data), Loxandrini (Costa et al. 2011), and are poorly developed in Licinini (Fig. 12J). At the same time, similar outgrowths are absent from the pupae of Nebriini, Carabini (Fig. 12O), Pogonini (Fig. 12P), Bembidiini, Zabrini (Fig. 12Q–S), Lebiini (Fig. 12T, U) (our data), Cychrini (Busato 2012), Trechini (Masne 1938), Masoreini (Valdes 2002), Brachinini (Frank et al. 2009), and Paussinae (Di Giulio et al. 2007). The outgrowths on the abdominal segments in Cicindelinae are only superficially similar to those in Galeritini, because they are placed dorsal to the spiracles and, therefore, are formed by tergites rather than pleurites. The presence or absence of these outgrowths is correlated neither to the type of feeding nor to the lifestyle of the larvae, nor to the site of pupation. As a result, we can assume that the presence (but neither the size nor the shape) of such outgrowths in the pupae can be considered as evidence of a relationship.

### Adaptive features of the immature stages

Oviposition in mud cells has been described in the ground beetle tribes Pterostichini: *Percus* (Bargagli 1874; Lumaret 1971; Kavanaugh 1998), *Abax* (Löser 1970, 1972; Brandmayr and Zetto-Brandmayr 1974; Brandmayr 1977), *Pterostichus* (Brandmayr 1977; Sasakawa 2011; our data), *Lesticus* (Habu and Sadanaga 1969); Platynini: *Anchomenus* (Dicker 1951; Kreckwitz 1978), Agonum (Olisares) (our data); Sphodrini: *Calathus* (Brandmayr and Zetto-Brandmayr 1979); Chlaeniini: *Chlaenius* (Riley 1884; Claassen 1919; Kazuo 1956); Lebiini: *Lebia* (Chaboussou 1939); Galeritini: *Galerita* (King 1919; the present paper); and Brachinini: *Brachinus* (King 1919; Erwin 1967; Saska and Honek 2004). According to some authors, mud cells protect the eggs not so much from desiccation as from fungal infections (Löser 1972; Brandmayr 1977), being observed in species inhabiting coastal or wet forests and meadows (Riley 1884; Claassen 1919; King 1919; Erwin 1967; Sasakawa 2011). At the same time, the placement of mud cells with eggs can vary significantly under different conditions. For example, females of different *Abax* species in forest habitats lay them both on the lower and upper surfaces of stones, while in the alpine meadows they locate mud cells exclusively on the lower, shaded surface, thereby protecting them from increased insolation and desiccation (Brandmayr and Zetto-Brandmayr 1979). Probably, females of *Brachinus crepitans* (Linnaeus, 1758) laying some eggs in mud cells in the open dry habitats can be accounted for by their protection from desiccation (Saska and Honek 2004). All this seems to indicate that this form of behavior could have repeatedly evolved in different taxa of ground beetles for various reasons. At least two reasons can be suggested for mud cells of *Galerita*: a very thin and vulnerable chorion, and a prolonged embryonic development.

The long development of the eggs and the first instar larvae attracts special attention. Normally formed larvae hatch from the eggs after 11–20 (mean = 13.2) days of development, while the first instar larvae successfully molted into the second instar always after 6–10 (mean = 7.8) days. In both cases, a more extended development almost always ends in failure. Thus, the duration of egg development is always longer than that of the first instar larva. On the one hand, this is in consequence to the protection of the egg with a mud cell. On the other hand, due to the embryonization of the development, a complexly organized larva with a complete set of antennomeres and palpomeres, paired claws, large urogomphi and a well-developed secondary chaetome emerges from the egg. The relatively quick development of the third instar larva is possibly a long-term consequence of such an embryonic development. In this case, the third instar larvae are developed only 1.35–1.7× longer than the second instars, whereas in most Carabidae the duration of their development is 2½–3× longer.

During the embryogenesis, a pleural organ, or pleuropodia/pleuropod, is formed on abdominal segment I of the larva in various insects (Fig. 9H). It is present in almost all Hemimetabola and performs a secretory function, participating in the metabolism and excretion (Polivanova 1982), as well as in the destruction of the serous membrane before molting (Slifer 1938; Louvet 1973; Konopova et al. 2020). In Holometabola, the pleuropodia are not always developed (see a review in Konopova et al. 2020), and in Coleoptera they are only known in a few groups, for example Dytiscidae (Niikura et al. 2017), Gyrinidae (Komatsu and Kobayashi 2012), and Cantharoidea (Ando and Kobayashi 1975; Kobayashi et al. 2003). Information concerning the functions of pleuropodia in beetle larvae is very scant. Only in Rhagophthalmidae its role in the destruction of the serous membrane has been confirmed (Kobayashi et al. 2003). Among Carabidae, the pleuropodia are only found in Carabini. In their first instar larvae just emerged from the egg, rudimentary pleuropodia are retained (Verhoeff 1917; Bengtsson 1927; Kirchner 1927; Hůrka 1971; Kobayashi et al. 2013). The preserved rudiments of pleuropodia in the larvae of Galeritini is only a second record of this structure in ground beetles. Although the function of pleuropodia has not been studied in detail, it seems important that pleuropodia are found in beetles with a high level of embryonization of their development. In Galeritini, it is a rather complexly organized larva with large appendages and a complicated chaetotaxy pattern that emerges from the egg. It is noteworthy that the larva of *Athemus suturellus* Motschulsky, 1860 (Coleoptera, Cantharidae), with its embryonic development lasting ca. ten days, has no pleuropodia (Fujiwara and Kobayashi 1987), while the larva of *Luciola cruciata* Motschulsky, 1854 (Coleoptera, Lampyridae), with a twice as long embryonic development, has them (Ando and Kobayashi 1975). Thus, we can hypothesize that the presence of pleuropodia is a prerequisite to an embryonization of the development, in which it is required to provide nutrition and the formation of a large and complexly organized larva.

All known Galeritini larvae (Sallé 1846; Candèze 1861; Hubbard 1875; van Emden 1942; Kawada 1959; Thompson 1979; Kirk 1980; Costa et al. 1988; Arndt and Drechsel 1998; Makarov 2002; Dajoz 2005; dos Santos 2012) show a set of striking features related primarily to their lifestyle. The contrast coloration, long legs and urogomphi, and well-developed eyes are characteristic of epigeobiont runners (Sharova 2008; Sharova and Makarov 2012). Due to the extremely long hind legs in Galeritini larvae, the apex of their abdomen does not touch the substrate during movement. Because segment X is very short and lacks secondary chaetae on the ventral surface (Fig. 9N’), it has lost the locomotor function typical of most larvae of ground beetles. Sternites of segment IX are also with fewer secondary setae than the preceding ones. The fusion and sclerotisation of abdominal sternites, starting with sternite IV, is another feature associated with an epibiotic lifestyle. This structure, together with a large number of secondary macrosetae, increases the protection of the abdomen, but reduces its flexibility. Similar, although somewhat less expressed morphological features of the abdomen, are found in the larvae of Loricerinae. The proportions of legs (distal parts are longer than proximal ones) and leg chaetotaxy (secondary chaetae are relatively thin and grouped in longitudinal rows) are also typical of epibionts.

The structure of the mouthparts of the Galeritini larvae is highly peculiar as well. The antennae, maxillae, labium, and, partly, forelegs are equipped with rows of thick and slightly curved setae with sharp apices and protruding tubercles at the base. Altogether, these chaetae and elongated head appendages form a trapping apparatus capable of capturing and holding small prey (Fig. 7B). In the third instar larvae, the distance between the distal groups of setae of half-divorced antennae (45° from the longitudinal body axis) is 4.3–5.4 mm (mean = 4.9 mm). Thereby the mandibles are small, the terebra in the proximal half show thin sharp teeth, a penicillus is absent, and the attachment area of the adductors of the mandible on the parietal sclerites is small. As a result, we can suggest that small (< 5 mm) mobile invertebrates, like springtails with a rather thin cuticle, seem to serve as the main diet for *Galerita* larvae in nature (Dajoz 2005). According to the available information, small arthropods and larvae of other beetles are the hunting objects for the larvae of *G. brasiliense* and *G. carbonaria* (Costa et al. 1988). Under laboratory conditions, Galeritini larvae can take different food like pieces of different Tenebrionidae larvae, small insects (cockroaches, aphids, termites, ants), as well as pieces of earthworms (Costa et al. 1988; dos Santos 2012, our data) and even veal (Schaupp 1878a).

### Taxonomic position of Galeritini

Van Emden (1942) was the first to consider *Galerita* as based on larval features within the “tribe Dryptini Bonelli, 1810”. The first diagnosis of the tribe Galeritini using larval features was formulated by Thompson (1979), later also confirmed by Arndt and Drechsel (1998) on the basis of the features shown by the *Trichognathus* larva. A comparison of the larvae of *G. ruficollis* with those of three unidentified *Galerita* species from Africa and Southeast Asia, as well as with the available literature data (Sallé 1846; Candèze 1861; Hubbard 1875; van Emden 1942; Kawada 1959; Thompson 1979; Kirk 1980; Arndt and Drechsel 1998; Costa et al. 1988; Bousquet 2010; dos Santos 2012), allows us to update the diagnosis of the tribe Galeritini.

The tribe Galeritini is characterized by a bifurcated protrusion on the frontale (Figs 7A, 8A–C), a ventral carina on the mandible (Fig. 7C), a sclerotized ligula, long trichobothria AN_4_, AN_5_, AN_7_ (Fig. 7A–D) (in *T. marginipennis*, only two of them are long), two short conical sensilla near the antennal sensory appendage (Fig. 7B), one or two spiniform setae UN_1_, UN_2_ (Fig. 9L), fused ventrites of abdominal segments IV–IX (Fig. 9J), apically truncated macrosetae and widened subcylindrical microsetae. The pupa has long and club-shaped outgrowths on the pleurites of abdominal segments II–VI (Fig. 11) (no pupae of any species of Dryptini are known yet).

A subapical position of the conical sensilla on the 4^th^ antennomere, very long macrosetae AN_4_ and AN_5_, the absence of a penicillus and gMX, very large MX_2_, MX_3_, MX_5_, and MX_6_, sclerotized ventrites of the thoracic segments, fused ventrites of abdominal segments VII–IX, long legs with a similar differentiation of setae in gTI and gFE, a medial articulation on tergite IX, as well as segmented urogomphi with additional bell-shaped sensilla are considered as the shared features both Galeritini and Dryptini larvae.

Some of these features can be explained as adaptations to moving on the surface of a substrate, with similar structures observed in larvae from other tribes of Carabidae as well. What seems important is that functionally similar results in various groups of ground beetles could have been achieved in different ways. For example, the mobility of the urogomphi can be due to articulation at their base or the connection of the left and right halves of abdominal tergite IX. The forward-directed head appendages can be both a hypertrophied nasale and a protrusion of the frontale. A sclerotized ventral surface of the abdomen can be gained by a thickened membrane or by fusion of sclerites. Eventually, all such cases indicate parallelisms (the adaptive effect is achieved in one way) or convergences (the adaptive effect is achieved in a different way), and we can use them to establish kinship. In our opinion, the similarity in the development (but to a varying degree) of the frontale protrusion, in the sclerotisation of the thoracic and abdominal ventrites, as well as in the medial articulation on abdominal tergite IX in the larvae of Galeritini and Dryptini prove to be evidence of sister relationships of these two tribes. The larvae of these tribes showing very long AN_4_ and AN_5_, the absence of compact gMX (differently sized setae evenly distributed on the stipes instead), and a complex of macrosetae on the maxilla can be considered as synapomorphies. The absence of a penicillus is not a unique feature, because it is also absent from the related Helluonini (Bousquet 2010), Anthiini (van Emden 1942), and Physocrotaphini (Makarov 1998), as well as from the unrelated Omophronini, Nebriini, Notiophilini, Ozaenini, and Orthogoniini. Thus, the monophyly of the subfamily Dryptinae Bonelli, 1810 seems to be well-supported.

Unfortunately, no larvae of Zuphiini are known yet. All published data are limited to the paper by van Emden (1942), who putatively assigned to Zuphiini one larva collected in French Guinea (now Guinea, Conakry) for the reasons of ”Obviously a very isolated type of Truncatipennes”. However, we can assert now that the features of this larva (unequal claws, the absence of a penicillus, a small retinaculum, a large antennal sensory appendage, protruding angles of the paraclypeus, etc.) are also known in different combinations in the larvae of Anthiini, Helluonini, and Physocrotaphini. Thus, the attribution of the larva described by van Emden (1942) to Zuphiini remains unproven. Therefore, we still refrain from discussing the relationships of Zuphiini with Dryptini and Galeritini based on larval traits. We can only agree that the larval features do not contradict the hypothesis that Anthiini, Helluonini, Physocrotaphini, Dryptini, and Galeritini belong to the same monophyletic lineage (Ober and Maddison 2008).

Traditionally, Galeritini and Dryptini, together with the related taxa, have been considered within the Truncatipennia, while in most cases the latter’s artificial composition is recognized. A reduced lacinia is the sole synapomorphy of the larvae of Truncatipennia (Arndt 1993, 1998). However, like any feature based on a reduction, homoplasy is highly probable to be the case. Indeed, a lacinia is also either absent from Ozaenini, Loricerini, Dyschiriini, Bembidiini, Trechini, Patrobini, Sphodrini, and Platynini or this can vary within the tribe, as in Elaphrini and Broscini. Considering ongoing new information on larval and pupal morphology, there seems to be a sound reason for returning to this question in the nearest future. We consider it highly important (see above) to take into account the similarly ensured mobility of the urogomphi, as well as the presence of outgrows of the pleurites in the pupa of Galeritini, Dryptini, and Licinini, like in Platynini, Chlaeniini, Pterostichini, and Harpalini. In other groups of Truncatipennia, the pleural area of the pupae is devoid of outgrowths, and the urogomphi, if mobile, are articulated at the base. Possibly the taxa that Erwin (1991) assigned to the supertribes Anthiitae and Zuphiitae are to be considered separately from the remaining Truncatipennia.
